# The Phytoplankton *Nannochloropsis oculata* Enhances the Ability of *Roseobacter* Clade Bacteria to Inhibit the Growth of Fish Pathogen *Vibrio anguillarum*

**DOI:** 10.1371/journal.pone.0026756

**Published:** 2011-10-28

**Authors:** Emilia Noor Sharifah, Mitsuru Eguchi

**Affiliations:** Graduate School of Agriculture, Kinki University, Nara, Japan; Argonne National Laboratory, United States of America

## Abstract

**Background:**

Phytoplankton cultures are widely used in aquaculture for a variety of applications, especially as feed for fish larvae. Phytoplankton cultures are usually grown in outdoor tanks using natural seawater and contain probiotic or potentially pathogenic bacteria. Some *Roseobacter* clade isolates suppress growth of the fish pathogen *Vibrio anguillarum*. However, most published information concerns interactions between probiotic and pathogenic bacteria, and little information is available regarding the importance of phytoplankton in these interactions. The objectives of this study, therefore, were to identify probiotic *Roseobacter* clade members in phytoplankton cultures used for rearing fish larvae and to investigate their inhibitory activity towards bacterial fish pathogens in the presence of the phytoplankton *Nannochloropsis oculata*.

**Methodology/Principal Findings:**

The fish pathogen *V. anguillarum*, was challenged with 6 *Roseobacter* clade isolates (*Sulfitobacter* sp. (2 strains), *Thalassobius* sp., *Stappia* sp., *Rhodobacter* sp., and *Antarctobacter* sp.) from phytoplankton cultures under 3 different nutritional conditions. In an organic nutrient-rich medium (VNSS), 6 *Roseobacter* clade isolates, as well as *V. anguillarum*, grew well (10^9^ CFU/ml), even when cocultured. In contrast, in a phytoplankton culture medium (ESM) based on artificial seawater, coculture with the 6 isolates decreased the viability of *V. anguillarum* by approximately more than 10-fold. Excreted substances in media conditioned by growth of the phytoplankton *N. oculata* (NCF medium) resulted in the complete eradication of *V. anguillarum* when cocultured with the roseobacters. Autoclaved NCF had the same inhibitory effect. Furthermore, *Sulfitobacter* sp. much more efficiently incorporated ^14^C- photosynthetic metabolites (^14^C-EPM) excreted by *N. oculata* than did *V. anguillarum*.

**Conclusion/Significance:**

Cocultures of a phytoplankton species and *Roseobacter* clade members exhibited a greater antibacterial effect against an important fish pathogen (*V. anguillarum*) than roseobacters alone. Thus, cooperation of *N. oculata*, and perhaps other phytoplankton species, with certain roseobacters might provide a powerful tool for eliminating fish pathogens from fish-rearing tanks.

## Introduction

*Nannochloropsis oculata*, a marine eukaryotic unicellular phytoplankton, is extensively used in the aquaculture industry. It grows in a wide salinity range and has significant nutritional value because of its high content of protein and polyunsaturated fatty acids, especially eicosapentaenoic acid [1]. *N. oculata* belongs to the class Eustigmatophyceae; its cells are spherical or slightly ovoid in shape and measure approximately 2–4 µm in diameter.

Phytoplankton cultures are widely used in the aquaculture industry for a variety of purposes. These cultures are described as “green water” because they contain high levels of phytoplankton species such as *Nannochloropsis* sp. and *Chlorella* sp. “Green water” is added to fish larvae tanks because it enriches zooplankton and provides indirect and direct nutrition to fish larvae. Moreover, green water reduces the transparency of rearing water, minimizing larval exposure to light which acts as a stressor. Phytoplanktons also improve water quality by reducing ammonium ion concentrations and increasing dissolved oxygen concentrations by photosynthesis. Notably, they also produce antibacterial substances that can prevent disease outbreaks [2], [3], [4], [5], [6].

Most aquaculturists produce green water using untreated natural seawater in open air. Green water modifies the bacterial composition of water [7]. For example, when *Nannochloropsis* sp. are introduced into a culture, 46% of the total bacteria grow actively, with the population being dominated by *Alphaproteobacteria* and the *Cytophaga-Flavobacterium* cluster [8]. The dominance of both bacterial groups is highly associated with increased production of cultured fish larvae [9].

A number of *Roseobacter* clade members (*Alphaproteobacteria*) suppress the growth of the devastating fish pathogen *Vibrio anguillarum* [10], [11], [12], [13]. Tropodithietic acid (TDA) produced by some *Phaeobacter* and *Ruegeria* isolates inhibits growth of *V. anguillarum* [11], [13], [14], [15], [16]. In addition, *Roseobacter* clade bacteria abundance was highly correlated with phytoplankton blooms [17]. As informative as these studies are, they are few and have left a number of questions unanswered, such as whether other antibiotics are produced in cocultures. We were inspired, therefore, to extend these studies in order to fully exploit the potential aquacultural benefits of the synergism between *Roseobacter* clade members and phytoplankton like *N. oculata*.

## Materials and Methods

### Culture Conditions

*N. oculata* was obtained from the Susami Fish Nursery Center, Kinki University, Japan. Cultures (10^6^ cells/ml) were started by transfer to freshly prepared phytoplankton culturing medium (ESM) containing artificial seawater (Nine Salt Solution, NSS) [18] and cultured for 7 days at 15°C. Each liter of ESM medium contained 12 mg NaNO_3_, 0.5 mg K_2_HPO_4_, 0.1 µg vitamin B_12_, 0.1 µg biotin, 10 µg thiamine HCl, 25.9 µg Fe-EDTA, 33.2 µg Mn-EDTA, 100 mg Tris (hydroxymethyl) aminomethane, and 2.5 ml of soil extract [19]. When the density (estimated daily using a Thoma blood-counting chamber, Hirschmann Techcolor) reached 10^8^ cells/ml in late log-phase, the culture was filtered as described below in the “*N. oculata* culture filtrate (NCF) and media preparation” section.

### Estimation of *Roseobacter* clade populations in *N. oculata* cultures

Ten-milliliter aliquots of *N. oculata* cultures were collected and fixed immediately with 20% paraformaldehyde-phosphate buffered saline (PBS) (pH 7.2) (final concentration, 2%). The fixed samples were filtered through a 0.2-µm polycarbonate filter for cell counts using the fluorochrome 4′, 6-diamidino-2-phehylindole (DAPI) [20]. *Roseobacter* numbers were estimated by fluorescence *in situ* hybridization (FISH) [21] using the 16S ribosomal RNA probe ROSEO536R [22]. Cells were counted using an epifluorescence microscope (BX-51; Olympus, Tokyo Japan) and UV (DAPI) and green (Cy3) excitation. FISH and DAPI double-positive bacteria were counted and designated as hybridized cells. *Roseobacter* abundance was expressed as the ratio of hybridized to total DAPI-stained cells. All experiments were performed at least in triplicate.

### Isolation and identification of *Roseobacter* clade members

Aliquots of *N. oculata* cultures were serially diluted 10-fold (up to 10^−6^) and spread onto VNSS agar (0.5 g yeast, 1 g trypticase peptone, 0.5 g glucose, 0.01 g FeSO_4_·7H_2_O, 0.01 g Na_2_HPO_4_·H2O, and 15 g Bacto Agar in 1 L artificial seawater) [18] and incubated in the dark at 20°C for 2–5 days. Beige, brown, or pink colonies were selected, because these colors are highly characteristic of the roseobacters [23]. Colonies isolated by 2 rounds of streaking were sub-cultured in VNSS. The *Roseobacter* were first identified by FISH, as indicated above, and by PCR.

The 16S rRNA gene was amplified by PCR using a universal primer, forward primer 27F (5′-AGAGTTTGATCMTGGCTCAG-3′), and reverse primer 1492R (5′-TACGGYTACCTTGTTACGACTT-3′). All PCR products were purified by ExoSAP-IT (USB Corporation) and sequenced by Solgent Co. Ltd. with the 16S rRNA gene primers 27F, 1492R, 518R (5′-CCAGCAGCCGCGGTAATACG-3′) and 785F (5′-GGATTAGATACCCTGGTA-3′). Sequences were tested with the BLAST program for matches to sequences deposited in GenBank, National Center for Biotechnology Information, USA (http://www.ncbi.nlm.nih.gov/genbank/index.html). Multiple sequences were simultaneously aligned using ClustalW and phylogenetic trees were generated using the neighbor-joining (NJ) method [24] with MEGA4 software [25]. The nucleotide sequences of the 16S rRNA genes determined in this study have been deposited in the DDBJ (DNA Data Bank of Japan) database under accession numbers AB607861 to AB607885.

### Bacterial culture conditions

We studied *Roseobacter* isolates that we obtained here from *N. oculata* cocultures and *Vibrio anguillarum* strain psh-9019 (*Gammaproteobacteria*), originally isolated from a diseased fish, *Plecoglossus altivelis* (Salmoniforms) [26], [27]. *V. anguillarum* strain psh-9019 (serotype J-O-1) causes vibriosis in freshwater and seawater fish [28]. Bacteria were cultured for at least 16 h with shaking (120 rpm) in VNSS liquid medium. After incubation, 1 ml of each bacterial culture was centrifuged at 8,000×*g* for 5 min and washed twice with sterile 3% NaCl.

### *N. oculata* culture filtrate (NCF) and media preparation

Three different types of media were used to compare the growth of roseobacters and *V. anguillarum* as follows: 1) VNSS, an organic nutrient-rich medium, for heterotrophic marine bacteria; 2) ESM, a phytoplankton culturing medium used for cultivating *N. oculata*; and 3) NCF, a medium containing substances excreted by *N. oculata* grown in ESM. To prepare NCF medium, *N. oculata* was cultured for 7 days until late log-phase and then centrifuged at 5,000×*g* for 15 min. The supernatant was passed through a GF/C filter (Whatman) and then sterilized by filtration through a 0.1 µm pore polypropylene syringe filter (Iwaki) and kept at 4°C. All experiments were carried out within 14 hours after filtration. Both ESM and VNSS media were sterilized by autoclaving at 121°C for 20 min.

### Effects of *Roseobacter* clade strains on the viability of *V. anguillarum*

*Roseobacter* strains RO3, RO7, R11, R16, R18, or R27, were each inoculated together with *V. anguillarum* strain psh-9019 into VNSS, ESM and NCF media at a final concentration of 10^4^ cells/ml in quadruplicate. Individual quadruplicate cultures of each strain in these same media served as controls. Aliquots (1 ml) were taken once daily for 7 days and serially diluted 10-fold. Ten microliters of these dilutions were added (5 drops per dilution) to VNSS agar plates using the drop plate method [29], incubated in the dark at 20°C for 3 days, and colonies were counted after incubation using a stereoscopic optical magnifier (Nikon model: 232063, 2× objective). *Roseobacter* and *V. anguillarum* colonies could be easily differentiated due to the differences in colony colors: cream-brown and white-gray, respectively.

### Uptake of compounds excreted by *N. oculata*

To determine whether *Roseobacter* clade strain RO3 and *V. anguillarum* strain psh-9019 were able to take up metabolites excreted by *N. oculata*, we followed the method of Berman [30] as modified by Kamjunke et al. [31]. Axenic *N. oculata* NIES-2145 (Microbial Culture Collection, National Institute for Environmental Studies, Japan) was cultured for 7 days in ESM medium (200 ml) until late log-phase. One milliliter of the undiluted axenic culture was spread onto ESM and VNSS agar media to check for bacterial contamination. NaH^14^CO_3_ (50 mCi/mmol; PerkinElmer Life & Analytical Sciences) was added (final concentration, 1 µCi/ml) to the *N. oculata* culture, which was then incubated for 14 h under fluorescent light (50 µmol photons/m^2^/s). The culture was then passed through a GF/C filter, and the filtrate (^14^C-EPM: ^14^C- labeled excreted photosynthetic metabolites) (10 ml) collected in sterile 15-ml tubes.

*Roseobacter* clade strain RO3 and *V. anguillarum* strain psh-9019 were grown and pre-cultured as described above in “Bacterial culture conditions.” Heat-killed (70°C for 45 min) *Roseobacter* clade strain RO3 and *V. anguillarum* strain psh-9019 served as controls. Both viable and control heat-killed organisms were added individually to 10 ml of *N. oculata* filtrate containing ^14^C-EPM. After 0 h,1 h, 3 h, and 6 h, 0.5 ml of each subsample was passed through 0.22-µm nitrocellulose membrane filters (Millipore), which were washed with 1 ml of 1 M HCl and dried in a ventilated chamber overnight to remove the remaining inorganic ^14^C. After drying, filters were inserted into 4-ml scintillation vials containing 3 ml Optiphase HiSafe 3 scintillator (PerkinElmer Life & Analytical Sciences), and radioactivity was counted in a liquid scintillation counter (LSC-5100, Aloka). Experiments were performed in quadruplicate.

### Heat stability of the EPM

*N. oculata* filtrates (NCF) were prepared as described in the “*N. oculata* culture filtrate (NCF) and media preparation” section above. After filtration, NCF were autoclaved at 121 °C for 20 minutes. The ability of *Roseobacter* clade strain RO3 to inhibit the growth of *V. anguillarum* in the presence of autoclaved NCF medium was determined as described above.

### Statistical analysis

The Shapiro-Wilk tests were used to test the null hypothesis that samples were acquired from a normally distributed population. Significant differences between control and individual samples of each medium were analyzed with independent samples using the Student *t*-test. The Mann-Whitney U test were used to evaluate data that were not normally distributed. Statistical analyses were done using StatPlus:mac 2009 (AnalystSoft Inc., USA).

## Results

### Estimation of *Roseobacter* clade populations in *N. oculata* cultures

*N. oculata* cell densities in late log-phase “green water” ranged from 2.3 to 6.3×10^6^ cells/ml (3 trials), and total bacterial counts were 1.3 to 3.8×10^6^ cells/ml (Table 1). *Roseobacter* clade bacteria estimated by FISH in *N. oculata* late-log cultures represented 11.4% to 13.2% of the total bacteria (Table 1).

**Table 1:** Estimation of *Roseobacter* clade populations in *N. oculata* cultures.

	*N. oculata* density (×10^6^ cells/ml)	Total bacteria count (×10^6^ cells/ml)	Proportion of *Roseobacter* clade bacteria (%)
Trial 1	6.3±1.6	3.8±0.6	11.4±1.9
Trial 2	6.1±0.6	3.1±2.9	12.8±1.3
Trial 3	2.3±1.9	1.3±3.2	13.2±1.6

± indicated SD (n = 3 for each trial).

### Isolation and identification of *Roseobacter* clade species

**Figure 1:**
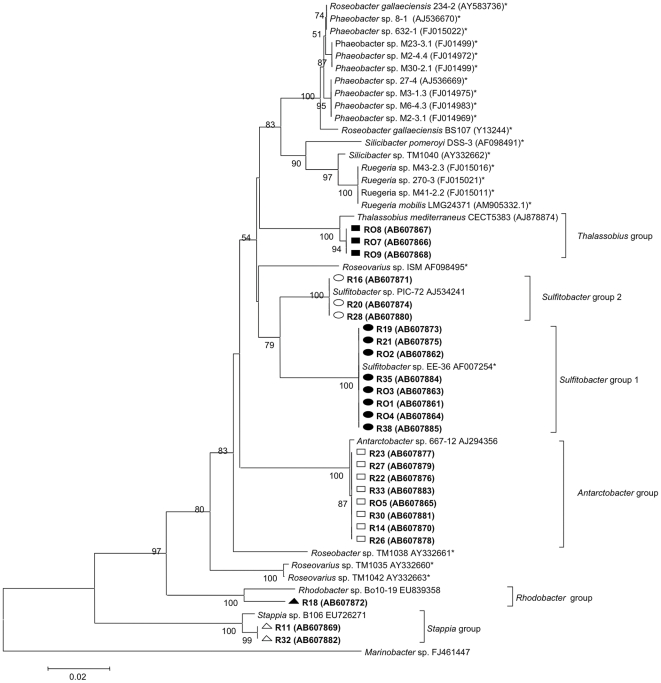
Phylogenetic tree of *Roseobacter* clade members isolated from *Nannochloropsis oculata* compared with potential probiotic species (*). Closed squares: *Thalassobius* group, open circles: *Sulfitobacter* group 2, closed circles: *Sulfitobacter* group 1, open squares: *Antarctobacter* group, closed triangles: *Rhodobacter* group, and open triangles: *Stappia* group. Numbers by the branches are bootstrap values from 1000 replicates (>50%). The scale bar indicates the number of base pair substitutions per nucleotide position. *Marinobacter* sp. was used as the outgroup. *Roseobacter* isolated in this study are in bold type.

Two-hundred forty-two bacterial strains were isolated from the *N. oculata* culture by the serial dilution method described above (Trial 3 in Table 1). Twenty-five strains were identified by FISH as *Roseobacter* clade bacteria. Their 16S rRNA genes were sequenced and shown to be related to the following *Roseobacter* clade genera: *Sulfitobacter* sp., *Antarctobacter* sp., *Thalassobius* sp., *Stappia* sp., and *Rhodobacter* sp. (Fig. 1). All 25 isolates could be assigned to the *Roseobacter* clade [32] and could be divided into 6 groups representing 5 genera (2 *Sulfitobacter* groups) (Fig. 1). Isolates RO3, RO7, R11, R16, R18, and R27 representing each group were tested for their abilities to inhibit *V. anguillarum* (Fig. 1 and Table 2).

**Table 2:** Phylogenetic sequences of culturable *Roseobacter* clade isolated from *N. oculata* culture.

Isolate	aAccession no.	Closest relative	Similarity (%)	Source of closest relative
RO3	AB607863	*Sulfitobacter* sp.	100	deep sea sediment
RO7	AB607866	*Thalassobius* sp.	98.1	seawater
R11	AB607869	*Stappia* sp.	98.8	deep seawater
R16	AB607871	*Sulfitobacter* sp.	100	seawater
R18	AB607872	*Rhodobacter* sp.	96.4	marine cyanobacteria
R27	AB607879	*Antarctobacter* sp.	99.6	dinoflagellates, *Alexandrium* spp.

a Accession number for sequences obtained in this study.

### Effect of *Roseobacter* clade strains on the viability of *V. anguillarum*

**Figure 2:**
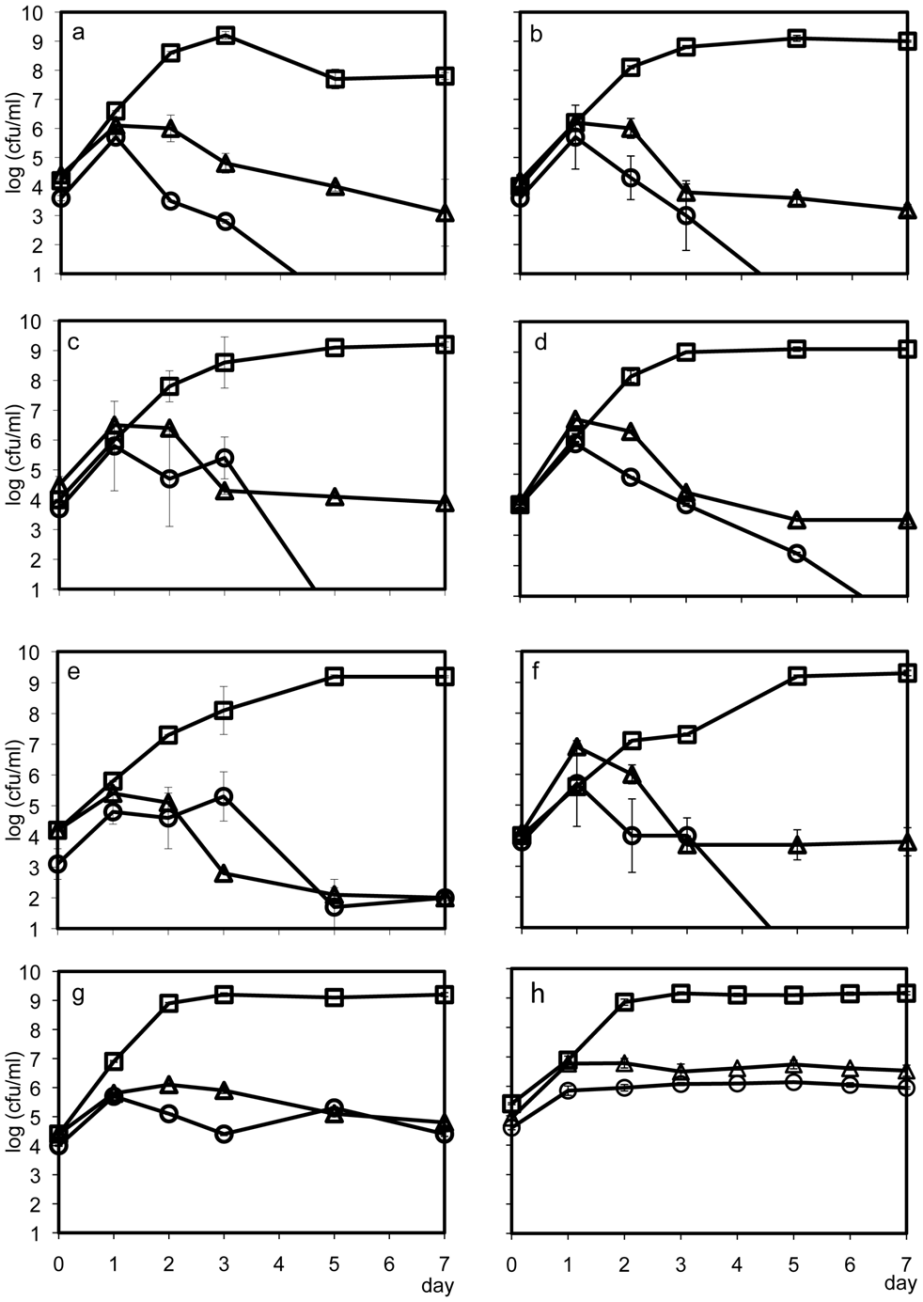
Cell densities of *Vibrio anguillarum* (a–g) and *Sulfitobacter* sp. RO3 (h) under 3 different nutritional conditions. *V. anguillarum* viable cell counts in the presence of a) *Sulfitobacter* sp. RO3, b) *Sulfitobacter* sp. R16, c)*Thalassobius* sp. RO7, d) *Antarctobacter* sp. R27, e) *Stappia* sp. R11, f) *Rhodobacter* sp. R18, and g) control (*V. anguillarum* only). Squares: VNSS (an organic nutrient-rich medium), triangles: ESM (phytoplankton culturing medium), and circles: NCF (medium containing excreted substances by *N. oculata*). (Error bars = standard deviation, SD).

In VNSS medium, which is rich in organic nutrients, the control *V. anguillarum* culture grew to 1.6×10^9^ CFU/ml (squares in Fig. 2g). Similar results were obtained in mixed cultures of *V. anguillarum* and *Roseobacter* clade bacteria (squares in Fig. 2b–f), except for *Sulfitobacter* sp. RO3 (squares in Fig. 2a) (P<0.05). In the presence of *Sulfitobacter* sp. RO3, *V. anguillarum* counts reached 1.6×10^9^ CFU/ml and then decreased 10-fold (squares in Fig. 2a). In VNSS medium, *Sulfitobacter* sp. RO3 grew to 2.3×10^9^ CFU/ml regardless of the presence of *V. anguillarum* (squares in Fig. 2h). All other roseobacters grew similarly to *Sulfitobacter* sp. RO3 in VNSS (data not shown).

In phytoplankton culturing medium, ESM, *V. anguillarum* cell counts in mixed cultures with 5 of 6 *Roseobacter* clade strains increased after 2 days from 1.3–3.2×10^4^ CFU/ml to 1.0–2.5×10^6^ CFU/ml, similar to controls (triangles in Fig. 2a–d and 2f). In the case of *Stappia* sp. R11, *V. anguillarum* counts only increased to 5.4×10^5^ CFU/ml (triangles in Fig. 2e). At the end of the experiment, the *V. anguillarum* control count in ESM was about 10^4^ CFU/ml (triangles in Fig. 2g). In stationary phase in ESM, *V. anguillarum* cell counts in mixed cultures were significantly lower than those in the controls, decreasing to 10^3^ CFU/ml (triangles in Fig. 2a–d and 2f) (P<0.05). *V. anguillarum and Stappia* sp. R11 counts were more than 10-fold lower than those of *V. anguillarum* cultured with the other roseobacters (∼1.0×10^2^ CFU/ml) (triangles in Fig. 2e) (P<0.05). Even though the coexistence of *Roseobacter* clade strains significantly decreased *V. anguillarum* numbers (in the stationary phase) in ESM medium, viable *V. anguillarum* persisted in mixed cultures for the entire experiment.

In NCF medium containing the substances excreted by *N. oculata*, control *V. anguillarum* CFUs were similar to those observed in ESM medium (circles in Fig. 2g). In contrast, when exposed to *Roseobacter* clade bacterial strains combined with substances excreted by *N. oculata*, all *V. anguillarum* were eradicated after 5 days of coculture (circles in Fig. 2a–d and 2f), except for *Stappia* sp. R11 (circles in Fig. 2e). When *V. anguillarum* was cocultured with *Stappia* sp. R11 in NCF, its colony counts were 1.0×10^2^ CFU/ml after 5 days and remained so until the end of the experiment (circles in Fig. 2e). In ESM and NCF (triangles and circles in Fig. 2h), *Sulfitobacter* sp. RO3 grew to 5.5×10^5^ and 6. 7×10^6^ CFU/ml, respectively, even in the presence of *V. anguillarum*. Other roseobacters showed the same growth patterns as *Sulfitobacter* sp. RO3 (triangles and circles in Fig. 2h) in ESM and NCF media when cocultured with *V. anguillarum* (data not shown).

### Uptake of compounds excreted by *N. oculata*

**Figure 3:**
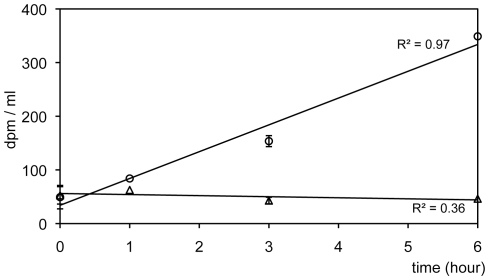
Uptake by *Sulfitobacter* sp. RO3 and *Vibrio anguillarum* of ^14^C-excreted photosynthetic metabolites (EPM) produced by *N. oculata* cultures. Circles: *Sulfitobacter* sp. and triangles: *V. anguillarum* (Error bars = SD).

^14^C-EPM uptake by *Sulfitobacter* sp. was significantly higher than that of *V. anguillarum* (circles in Fig. 3). There was a direct relation between time and nutrient uptake values for ^14^C-EPM by *Sulfitobacter* sp. (r^2^ = 0.97) for the 6 h of incubation. In contrast, there was no significant ^14^C-EPM uptake by *V. anguillarum* (triangles in Fig. 3). Furthermore, there was no significant difference in bacterial size and counts for both bacterial species during the incubation (data not shown). These findings provide a basis for establishing a potential inhibitory mechanism that operates in these mixed cultures.

### Heat stability of the EPM

**Figure 4:**
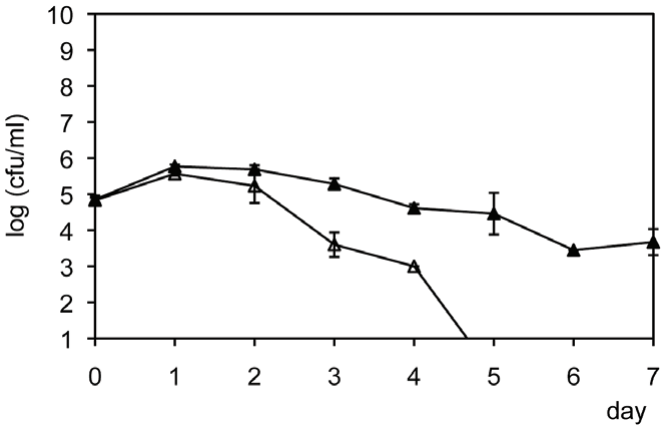
Cell densities of *V. anguillarum* in autoclaved NCF medium. Closed triangles: *V. anguillarum* only (control), and open triangles: *V. anguillarum* in the mix culture of *Sulfitobacter* sp. (Error bars = SD).

To gain some insight into the properties of the excreted by *N. oculata*, we tested the ability of autoclaved NCF medium to mediate the growth inhibition. This treatment had no detectable effect (Fig. 4).

## Discussion

Of all the bacterial genera present in marine environments, the *Roseobacter* clade is one of the most abundant. Here, we found that *Roseobacter* clade bacteria composed approximately 11.4–13.2% of the bacteria in indoor *N. oculata* cultures that were designed to simulate outdoor cultures at actual aquaculture production sites (Table 1). These values are comparable to *Roseobacter* levels in coastal seawater, (<1–25%) [17], [33], [34]. Among the culturable bacteria in the *Roseobacter* clade, *Phaeobacter* spp., *Silicibacter* sp. TM1040, *Sulfitobacter* sp. EE36, *Roseobacter* sp. TM1038, and *Roseovarius* spp. have been reported to possess potentially probiotic properties [11], [13], [15], [17] (Fig. 1 of the present study). Fig. 1 (in bold) shows that none of the *Roseobacter* clade species isolated from *N. oculata* cultures studied here represented known, potentially probiotic bacteria, except for *Sulfitobacter* sp. Our new isolates represent 5 (*Sulfitobacter* sp., *Antarctobacter* sp., *Thalassobius* sp., *Stappia* sp., and *Rhodobacter* sp.) of the 52 *Roseobacter* clade genera (Fig. 1). They might be useful as probiotics for treating fish infected with pathogenic bacteria such as *V. anguillarum*.

Adding phytoplankton to fish larvae tanks increases larval survival [3], [4], [5], [9] by inhibiting growth of pathogenic bacteria. This process could be mediated by at least 2 possible mechanisms. One involves either nutrient uptake preferences or nutrient competition, or both, by bacteria. The second, and by far more complex, entails direct interaction among microbes such as phytoplankton and pathogenic bacteria, probiotic bacteria and pathogenic bacteria, and phytoplankton-probiotic bacteria and pathogenic bacteria.

Regarding competition for nutrient uptake, *Roseobacter* clade abundance in coastal seawater correlates with the release of organic substances from natural phytoplankton blooms such as dimethylsulfoniopropionate (DMSP) [35], [36] and amino acids [37]. *N. oculata* may also excrete some substances like DMSP or amino acids that more optimally support the growth of *Roseobacter* clade bacteria. Our metabolic studies revealed that *V. anguillarum* did not appreciably incorporate ^14^C-labeled excreted metabolites by the phytoplankton, in contrast to *Sulfitobacter* sp. (Fig. 3). Furthermore, plate counts of *V. anguillarum* in NCF medium at the end of the experiment were similar compared to those in ESM medium (Fig. 2g). In the case of *Roseobacter* clade bacteria, however, their plate counts in NCF were 10-fold higher than that in ESM (triangles and circles in Fig. 2h). Alonso et al. [38] reported that in a low nutrient environment, *Roseobacter* clade bacteria were the main glucose consumers followed by *Gammaproteobacteria*. In other experiments not presented here, we observed a similar nutrient uptake pattern of ^14^C-labeled glucose by *Sulfitobacter* sp. and *V. anguillarum*. This indicates that *Roseobacter* clade bacteria more efficiently scavenge small amounts of organic nutrients under the oligotrophic conditions mimicked by ESM and NCF media. ESM medium contains soil extract and vitamins as the only supplemental organic nutrients. Thus, due to their more efficient nutrient-scavenging system, it is likely that *Roseobacter* clade bacteria will win the competition for nutrients over *V. anguillarum*. This would account for the poorer survival of *V. anguillarum* after reaching stationary phase in ESM medium (triangles in Fig. 2a–f). In contrast, *V. anguillarum* coexisted with *Roseobacter* clade bacteria in VNSS medium did not show such a decrease in CFU (squares in Fig. 2a–f). This might have occurred because there was either no apparent competition for nutrients or specific nutrient selectivity.

Regarding the second mechanism described above involving complex intercellular interactions, there was no direct inhibition of fish pathogens by phytoplankton, in contrast to other findings [39], [40]. As there was no difference in the viabilities of *V. anguillarum* between ESM and NCF (Fig. 2g), we concluded that *N. oculata* did not directly inhibit *V. anguillarum*'s viability. In contrast, the diatom *Skeletonema costatum* and the macroalga *Ulva clathrata* produce organic compounds that inhibit *V. anguillarum* [39], [40].

We believe that direct growth inhibition of bacterial fish pathogens by *Roseobacter* clade bacteria [10], [11], [12], [13], [41] may explain our findings (Fig. 2a–f). *Roseobacte*r clade bacteria benefit scallop [42] and turbot larvae [43] propagation by eliminating bacterial fish pathogens. Many studies have shown that cell densities of 10^6^–10^9^ CFU/ml *Roseobacter* clade bacteria were necessary to reduce the population of bacterial fish pathogens by approximately 10-fold [11], [12], [13], [16]. In the present study, in ESM medium, probiotic bacteria that grew to the highest density of 10^5^ CFU/ml directly inhibited *V. anguillarum* (triangles in Fig. 2a–f). This cell density, 10^5^ CFU/ml, is lower than those reported by others [11], [12], [13], [16].

Biofilm formation under static culture conditions enabled the bacteria *Phaeobacter* spp., *Silicibacter* sp. TM1040, *Sulfitobacter* sp. EE36, *Roseobacter* sp. TM1038, *Roseovarius* spp., and *Pseudoalteromonas* spp., to produce antibacterials against *V. anguillarum* [10], [13], [15], [44]. Tropodithietic acid (TDA) was reported to be the antibacterial compound produced by *Phaeobacter* spp., *Silicibacter* sp. and *Ruegeria* sp. [13], [15]. Static conditions and brown pigments were among the characteristics that correlate with TDA production [13], [15]. *Roseobacter* isolates in this study may produce antibacterial compounds other than TDA as the isolates were cultured in shaking conditions and did not produce brown pigment, although they exhibited antibacterial activity towards *V. anguillarum*. Shaking cultures more closely approximate actual conditions in an aquaculture facility.

Many studies have only focused on probiotic bacterial interactions with bacterial fish pathogens including *V. anguillarum* [10], [11], [12], [13], [14], [15], [16]. D′ Alvise et al. [16] observed eradication action of *V. anguillarum* by the probiotic bacteria, *Ruegeria* M43-2.3 (static culture) and *Phaeobacter* M23-3.1 (shaken and static cultures). Interestingly, our study demonstrated that shaken *Roseobacter* cultures were capable of killing *V. anguillarum* completely only in the presence of substances excreted from phytoplankton (circles in Fig. 2a–e), and none of them belonged to the *Phaeobacter* sp. group as observed by others [16] (Fig. 1).

The inhibitory activities of *Roseobacter* clade members *Sulfitobacter* sp., *Thalassobius* sp., *Rhodobacter* sp., and *Antarctobacter* sp. (but not *Stappia* sp.) against *V. anguillarum* were markedly affected by heat-stable substances excreted by *N. oculata* (circles in Fig. 2a–d and 2f) (Fig. 4). *N. oculata*, *N. granulata*, *N. oceanica*, *and N. salina* produce putrescine, a heat stable polyamine [45], [46]. Moreover, *N. oculata* CCMP525 produce low molecular weight signaling molecules similar to acyl-homoserine lactones, which are produced by bacteria for cell-to-cell communication systems that regulate gene expression [47]. Acyl-homoserine lactone analogs are heat stable [48]. These heat stable compounds may have been excreted by *N. oculata* in this study and could have acted as signaling molecules for communicating with *Sulfitobacter* sp. RO3 resulting in inhibition of *V. anguillarum*'s growth. These results showed that phytoplankton cultures used as “green water” for producing fish larvae likely play an important role in enhancing the inhibitory effect of *Roseobacter* clade bacteria against *V. anguillarum*. We also observed similar inhibitory effects of other marine microalgae used for aquaculture, such as a marine *Chlorella* sp. filtrate (data not shown).

In our recent studies, a microcosm experiment employing *N. oculata* cultures showed that both culturable and non-culturable *Vibrio* species pathogenic for fish, decreased when *N. oculata* started to grow exponentially [49]. Furthermore, an in vivo experiment we proved that a high abundance of *Alphaproteobacteria* in *N. oculata* cultures is the key factor for high survival and growth of fish larvae [9]. To our knowledge, the present study is the first to report that *Sulfitobacter* sp., *Thalassobius* sp., *Stappia* sp., *Rhodobacter* sp., and *Antarctobacter* sp. isolated from phytoplankton cultures could interfere with the growth of *V. anguillarum* and that *Roseobacter* clade bacteria in concert with a phytoplankton exhibited an enhanced antibacterial effect. *Roseobacter* clade bacteria were better at competing for nutrients or as antibacterial agents. Thus, our research provides compelling evidence that phytoplankton cooperating with certain roseobacters provides aquaculturists with a powerful tool for controlling bacterial fish pathogens.
